# Single-Trial fMRI Decoding of 3D Motion with Stereoscopic and Perspective Cues

**DOI:** 10.1523/JNEUROSCI.0044-25.2025

**Published:** 2025-04-22

**Authors:** Puti Wen, Lowell W. Thompson, Ari Rosenberg, Michael S. Landy, Bas Rokers

**Affiliations:** ^1^Psychology, New York University Abu Dhabi, Abu Dhabi, UAE; ^2^Department of Neuroscience, Perelman School of Medicine, University of Pennsylvania, Philadelphia, Pennsylvania 19104; ^3^Department of Neuroscience, School of Medicine and Public Health, University of Wisconsin-Madison, Madison, Wisconsin 53705; ^4^Department of Psychology and Center for Neural Science, New York University, New York, New York 10003

**Keywords:** 3D motion, binocular vision, fundus of the superior temporal sulcus, middle temporal area, MT complex

## Abstract

How does the brain process 3D motion? We focused on the human motion complex (hMT+), extending insights from monkey studies. Using 3D-motion stimuli containing perspective and/or stereoscopic cues, we investigated the hierarchy within the motion complex in humans of both sexes to understand the neural mechanisms underlying motion perception. On each trial we decoded 3D motion direction (toward/away) based on the BOLD response in primary visual cortex (V1), and regions within hMT+ including the middle temporal (MT) and medial superior temporal (MST) areas, and the fundus of the superior temporal sulcus (FST). We found that 3D-motion direction could be reliably decoded from all four areas but accuracy depended on cue content. MT and FST showed greatest decoding accuracy with perspective and stereoscopic cues, respectively. Decoding of motion direction in V1 and MST could be explained by retinotopic biases in the BOLD response to motion stimuli. MT and FST were less impacted by such biases. We also identified significant behavioral differences between participants: some were proficient at using stereoscopic cues and others performed near chance. Good behavioral performance with stereoscopic cues was accompanied by better decoding performance in FST but not MT. A control experiment that eliminated 3D motion percepts for stereoscopic stimuli, but not perspective stimuli, revealed that unlike MT, decoding accuracy in FST was influenced by perceptual components of 3D motion. Our findings support that MT and FST play distinct roles in the analysis of visual motion and are key in the transformation of retinal input into perceptual report.

## Significance Statement

Visual motion is processed in a hierarchy of regions in the primate brain. In humans, the hMT+ complex contains homologs of nonhuman primate (NHP) middle temporal (MT) and medial superior temporal (MST) areas. hMT+ was recently found to include a third subdivision consistent with the fundus of the superior temporal sulcus (FST) in NHPs. Here, we show that human FST and MT, like their NHP counterparts, are functionally distinguishable based on the representation of 3D motion. We find a perceptual representation of 3D motion in human FST, but not MT, that is distinct from the patterns of motion found on the retinae. This work shows how the human visual motion-processing network extends beyond MT to represent complex, perceptual motion signals.

## Introduction

Motion is processed hierarchically in the primate visual pathway. Primary visual cortex (V1) and the middle temporal (MT) area are two extensively studied sites in this pathway. Monkey studies have demonstrated selectivity for direction, speed, and binocular disparity in V1 (Orban et al., 1986; Prince et al., 2000) and MT (Maunsell and Van Essen, 1983a,b). Despite some overlap in functional properties, a hierarchical relationship is evident. For example, MT receives feedforward input from V1 (Anderson et al., 1998), MT receptive fields are almost tenfold larger than those in V1 (Albright and Desimone, 1987), and evidence suggests a two-stage process in which V1 neurons compute 1D local component motion and some MT neurons integrate the input of a population of V1 neurons to estimate 2D pattern motion (Movshon et al., 1985; Simoncelli and Heeger, 1998; Rust et al., 2006).

While the processing of 2D retinal motion is well characterized (Born and Bradley, 2005), it is complicated to translate this knowledge to the domain of 3D object motion. Some studies have suggested that MT neurons might exhibit 3D motion selectivity (Czuba et al., 2014; Sanada and DeAngelis, 2014), while others suggested that the apparent 3D motion tuning of many MT neurons can be explained based on joint 2D motion and static disparity sensitivity (Maunsell and Van Essen, 1983b). This is supported by a recent study demonstrating that many MT neurons' responses reflect 2D direction selectivity and ocular dominance (Thompson et al., 2023). This divergence suggests it would be useful to explore representations of 3D motion downstream of MT.

One region that emerges as a promising candidate is the fundus of the superior temporal sulcus (FST). In monkeys, neuroimaging (Héjja-Brichard et al., 2020) and electrophysiological (Thompson et al., 2023) studies have implicated FST in 3D motion processing. FST also has several distinct characteristics. Unlike MT (Qian and Andersen, 1994), FST contains neurons whose responses to locally opponent motion are not suppressed (Rosenberg et al., 2008). This could be helpful for estimating 3D motion, for example, when parsing the movement of surfaces at different depth planes to judge traffic at a busy intersection. FST's involvement in processing looming objects (Cléry et al., 2020), 3D structure from motion (Vanduffel et al., 2002; Mysore et al., 2010), and action-related vision (Nelissen et al., 2006) further differentiate it from nearby regions.

Here we use fMRI to test whether a hierarchical relationship between hMT and FST exists in the human motion-processing stream. Similar properties to monkey MT have been identified in the human MT complex, including the ability to decode motion direction (Kamitani and Tong, 2006; Serences and Boynton, 2007; Wang et al., 2014), motion adaptation (Huk et al., 2001; Rokers et al., 2009), coherence-dependent amplitude modulation (Rees et al., 2000), motion opponency (Heeger et al., 1999), and a columnar organization (Zimmermann et al., 2011). The involvement of hMT+ and potential areas downstream in 3D motion perception has also been supported by human neuroimaging studies (Likova and Tyler, 2007; Rokers et al., 2009; Wen et al., 2023). Recently, a human homolog of FST was localized anterior and ventral to hMT and medial superior temporal (MST; Wen et al., 2025). However, the specific contributions of MT and FST to 3D motion processing are unclear.

The current paper therefore addresses significant issues regarding motion cues, subdivisions of the motion complex, and decoding methods. First, we incorporate perspective cues (Thompson et al., 2021) in addition to previously studied stereoscopic cues. This approach reflects the significance of perspective cues to 3D motion perception (Thompson et al., 2019; Fulvio et al., 2020). Second, rather than treating hMT+ as a single region, we compare MT and FST and discuss other areas within the network (namely, V1 and MST) to clarify inconsistencies in the literature. Last, we leverage single-trial fMRI decoding instead of trial-averaged decoding to correlate BOLD responses with behavior as, paraphrasing others, nothing in neuroscience makes sense except in the light of behavior (Hirsch, 1986).

## Materials and Methods

### Observers

Ten healthy observers (four males, aged 23–49 years) with normal or corrected-to-normal vision participated in this study. Each participant provided written informed consent. All observers scored five or higher (70 s of arc or better) on the Randot Circles Stereotest (Stereo Optical). The study was conducted at New York University Abu Dhabi, where each observer took part in one scanning session for the main experiment and an additional session for the control experiment. Each scanning session lasted 1.5 h. The experiments were approved by the University Committee on Activities Involving Human Subjects at New York University and New York University Abu Dhabi.

### Visual stimulus—main experiment

We generated the stimuli on a Macintosh computer using MATLAB 9.2 (The MathWorks) and the Psychophysics Toolbox extensions (Brainard, 1997; Pelli, 1997; Kleiner et al., 2007). Stimuli were displayed using a ProPixx DLP LED projector (VPixx Technologies) with a screen resolution of 1,920 × 1,080 pixels and a refresh rate of 120 Hz. The projector was positioned at the back of the scanner, projecting onto a rear projection screen (width, 38.5 cm; viewing distance, 88 cm).

**Figure 1.**
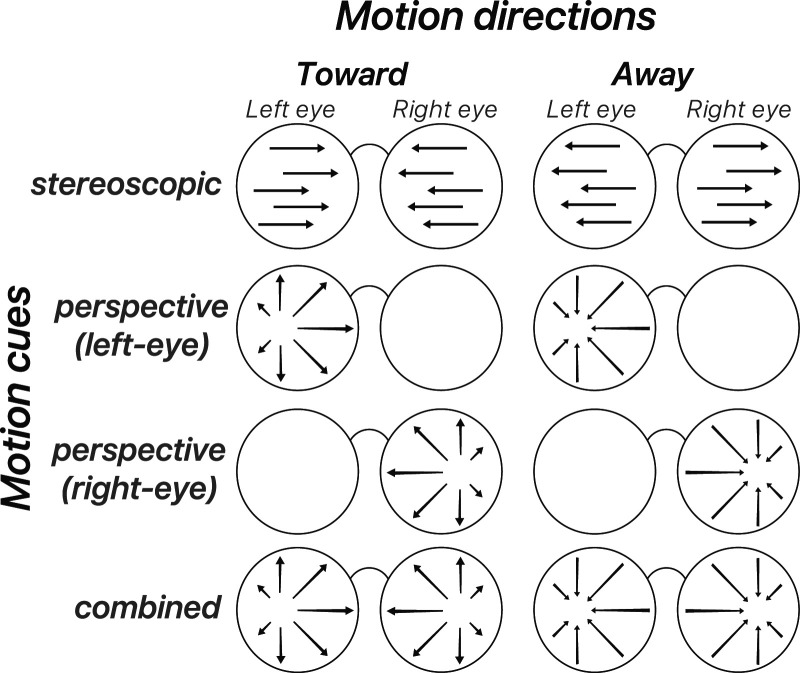
Visual stimulus design for the main experiment. Screen-projected vector fields of the stimulus for each motion direction and for each cue condition for the left and the right eye.

The stimuli, adapted from Thompson et al. (2021), included four conditions: the stereoscopic-cue condition, the two perspective-cue conditions (left-eye and right-eye conditions), and the combined-cue condition (Fig. 1). In the combined-cue condition, motion direction was signaled by both perspective and stereoscopic cues. Perspective cues included optic flow and changes in stimulus element density and size produced by projective geometry. In the stereoscopic-cue condition, motion direction was signaled by changing disparity and interocular velocity differences. In the stereoscopic-cue condition, coherent 2D motion signals also existed within each retina. In the perspective-cue conditions, only one eye was presented with the stimulus, while the other eye viewed a blank background and fixation point, preserving the perspective cues sufficient to distinguish toward and away motion directions. Example screenshots and videos of each stimulus type are available in Movie 1.

At the fixation plane, dots were 0.16° in diameter. In the perspective-cue and combined-cue conditions, the dot size changed with motion toward or away from the observer, whereas in the stereoscopic-cue condition, the dot size remained constant. The stimuli consisted of 80 black and white dots moving toward or away from the observer within a circular aperture with an outer radius of 8° and an inner radius of 0.25°. The aperture background was gray, and the area outside was filled with 1/*f* noise at the plane of fixation, identical for both eyes.

Dots moved at ∼51.83 mm/s in world units, and 1.2°/s in retinal units, with disparity changing at a speed of 2.4°/s. Each condition had two motion directions (toward and away), resulting in eight motion types. Each type was repeated five times per run, each trial lasted 1 s followed by an 8 s offset, and participants completed 10 runs. Participants performed a motion-discrimination task, indicating the perceived motion direction (toward or away) by pressing a button following the onset of each trial.

### Vertical control experiment

**Figure 2.**
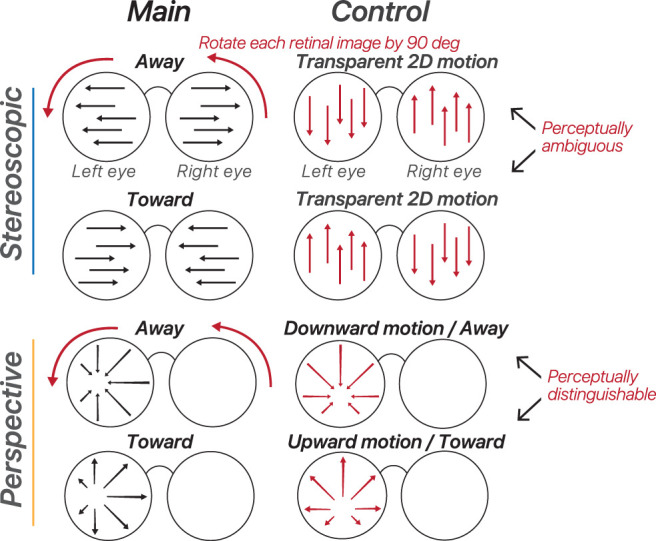
Visual stimuli for the control experiment. Each eye's retinal image was rotated by 90°, which creates transparent 2D motion for the stereoscopic condition. For the perspective-cue condition, the rotated control stimulus creates net 2D retinal motion as well as 3D motion through the perspective cues.

To control for potential confounding variables, participants took part in a control experiment using rotated stimuli. In the stereoscopic-cue condition for the control, each eye's retinal image was rotated by 90°, converting the perception—typically indicating movement toward or away from the observer—into transparent 2D motion (Fig. 2). This adjustment meant that one eye saw upward motion, while the other saw downward motion. Stimulus parameters, such as speed, luminance, and aperture, were consistent between the main and control experiments. The primary difference was in motion perception for the stereoscopic-cue stimuli: the main experiment elicited 3D motion percepts, whereas the control generated transparent 2D motion percepts. This experimental design ensured that any discrepancies in decoding accuracy for a specific region of interest (ROI) were due to the altered motion percept.

If an ROI showed a difference in decoding accuracy between the two experiments, it suggested that the ROI's decoding was not solely based on lower-level visual properties. Conversely, if there was no variation in decoding performance, it might indicate that the main experiment's decoding results could be attributed to lower-level stimulus properties rather than the observer's perceptual interpretation. The vertical control experiment in our previous study (Wen et al., 2023) revealed that the control manipulation slightly reduced overall decoding accuracy across the visual cortex. This decrease was only significant in regions involved in 3D motion processing, such as hMT+ and V3A (Wen et al., 2023). The observed reduction in decoding accuracy in these regions aligns with their known roles in encoding motion percepts, which were disrupted by the control manipulation. The idea was to compare the relative changes in decoding accuracy between different ROIs and conditions rather than focusing on absolute changes. Examining relative differences allows for the identification of region-specific effects that might be masked when only absolute changes are considered.

The vertical control experiment was also conducted for the perspective-cue condition from the main experiment. However, this manipulation preserved the perspective cues; thus no change in decoding accuracy was expected.

### MRI data acquisition and ROI identification

MRI data were acquired on a Siemens Prisma 3 T full-body MRI scanner (Siemens Medical Solutions) using a 64-channel head coil. For each observer, a T1-weighted anatomical scan was acquired (TR, 2,400 ms; TE, 2.22 ms; flip angle, 8°; 0.8 mm isotropic voxels). This anatomical volume was used for white/gray matter segmentation and coregistration with the functional scans. T2*-weighted functional scans were acquired using an echoplanar imaging sequence (TR, 1,000 ms; TE, 38 ms; flip angle, 68°; multiband factor, 4; matrix size, 104 × 104; 2 mm isotropic voxels; 68 slices). For each functional scan, we collected 360 volumes with full brain coverage, and an extra 10 s was discarded at the beginning of a scan.

**Figure 3.**
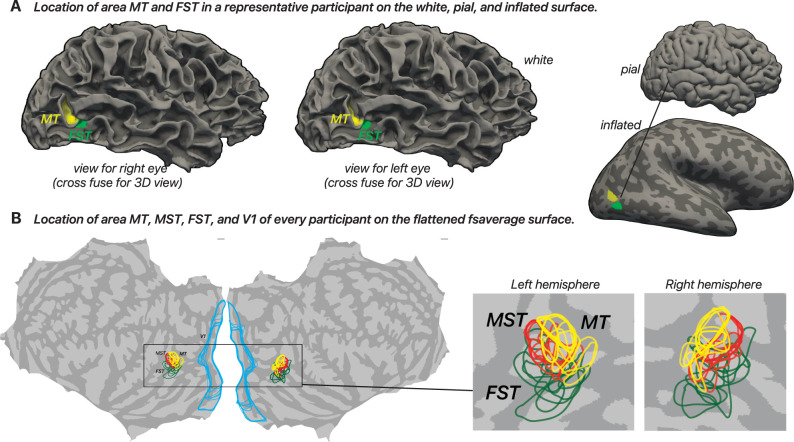
Surface visualization of MT, MST, FST, and V1 ROIs. ***A***, MT and FST in a representative participant’s right hemisphere, displayed from a lateral view on the white matter surface for stereoscopic viewing (left- and right-eye views for cross-fusion), and on the pial and inflated surfaces of the same subject. ***B***, Individual ROIs from all participants projected onto the fsaverage flattened cortical surface. Each colored outline represents one participant’s ROI, MT (yellow), MST (red), FST (green), and V1 (blue).

We defined ROIs using dedicated localizers and mapping methods. FST was identified with a disparity-defined dynamic random-dot stereogram that was distinct from the main experiment. The localizer contained changing disparity cues only and no coherent 2D motion signals (Wen et al., 2025). MT and MST boundaries were delineated using a 2D motion localizer and pRF-mapping results (available for 6 of the 10 participants); V1 was also identified using pRF results. For the remaining four participants, V1, MT, and MST were identified using the Glasser atlas (Glasser et al., 2016). While functional and atlas-based definitions are not always identical, previous work (Wen et al., 2025) suggests that MT/MST show less variability between these methods compared with FST. We also ensured that there were no overlapping vertices between identified ROIs. We show the location of every individual MT, MST, FST, and V1 ROI across participants, projected onto the fsaverage flat map, along with example views from a single participant on white, pial, and inflated surfaces in Figure 3. For detailed documentation of our mapping methods, see the GitHub repository: https://github.com/raniaezzo/DrawingROIs.

### fMRI preprocessing and statistical analysis

All scans were formatted according to the Brain Imaging Data Structure guidelines format (Gorgolewski et al., 2017). We employed the fMRIPrep pipeline (version 20.2.1) to perform motion correction, spatial normalization, and coregistration of functional and anatomical scans (Esteban et al., 2019). This pipeline also transformed our data into fsnative space, mapping the data onto the cortical surface using vertices instead of voxels. All further analyses were carried out on this surface data.

The resulting data in fsnative space had dimensions of 360 TR by the number of vertices. We preprocessed the data following the methods described in a previous single-trial decoding study (van Bergen and Jehee, 2021). This included applying a high-pass filter (1 cycle/40 s cutoff), regressing out motion and global signals, and *z*-normalizing the time-series across each *n*^th^ TR after the onset of each trial. To account for hemodynamic lag and obtain a single response amplitude for each vertex on each trial, we averaged the sixth to ninth *z*-scored values.

After preprocessing, the data had dimensions of 40 by the number of vertices, with each row representing a separate trial. This was done separately for each of the 10 runs, resulting in a final data size of 400 trials by the number of vertices per participant. Each participant had 50 trials per motion direction (toward vs away) per cue condition (two perspective-cue conditions, one stereoscopic-cue condition, and one combined-cue condition).

We used a support vector machine from MATLAB's *fitcsvm* function to decode the direction information from the trial-wise response amplitudes. The data were randomly split into a 10:90 test:train ratio, balanced across directions and bootstrapped 5,000 times. A random-chance decoding distribution was obtained by shuffling the training labels. This analysis was run separately for each ROI, for each cue condition, and for each participant.

## Results

### Single-trial decoding of 3D motion direction

**Figure 4.**
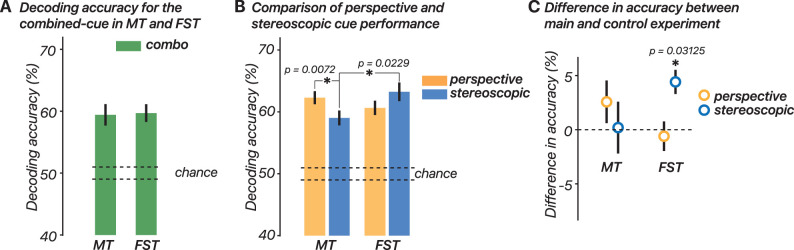
Single-trial decoding accuracy. ***A***, Decoding accuracy for the combined-cue stimulus in MT and FST. Bars represent mean decoding accuracy for the combined-cue condition in each ROI with error bars indicating the standard error of the mean (SEM) across participants (*n* = 10). The two dashed lines indicate the SEM of the chance distribution from decoding using randomly shuffled labels. ***B***, Comparison of perspective- and stereoscopic-cue performance in MT and FST. Bars represent mean decoding accuracy for perspective and stereoscopic cues, with error bars indicating SEM (*n* = 10). ***C***, Difference in accuracy between the main and control experiments (rotated stimulus). Points represent the difference in decoding accuracy between the main experiment and a control experiment with a rotated stimulus, with error bars indicating SEM across participants (*n* = 7). Unless otherwise specified, the perspective-cue results represent the average performance of the two perspective-cue conditions (left-eye only and right-eye only).

*Comparison of perspective- and stereoscopic-cue performance in MT and FST*. We reliably decoded the presented 3D motion directions (toward vs away) from MT and FST in all participants (*n* = 10) on a trial-by-trial basis (Fig. 4*A*) in the combined-cue condition. While there were no differences in decoding performance between ROIs in the combined-cue condition (Fig. 4*A*), distinct patterns emerged when the perspective- and stereoscopic-cue conditions were analyzed separately (Fig. 4*B*). A linear mixed-effect model was fit to the data with participant as a random effect, testing for an interaction between cue type (perspective vs stereoscopic) and ROI (MT vs FST). The model revealed a significant interaction between cue type and ROI (*t*_(36)_ = 2.95; *p* = 0.0055). Post hoc comparisons showed that in the stereoscopic-cue condition, FST had significantly greater accuracy than MT (*t*_(18)_ = 2.49; *p* = 0.023). Additionally, within MT, the perspective-cue condition showed significantly greater accuracy compared with the stereoscopic-cue condition (*t*_(18)_ = −3.03; *p* = 0.0072). In the perspective-cue condition, there was no significant effect of ROI on accuracy (*t*_(18)_ = −1.36; *p* = 0.19), and in FST, there was no significant effect of the cue type on accuracy (*t*_(18)_ = 1.52; *p* = 0.15). These results indicate that decoding in MT relies more on perspective cues than stereoscopic cues, while decoding in FST relies more on stereoscopic cues compared with MT.

*Difference in accuracy between main and control experiments*. Seven of the original ten participants took part in a control experiment with a rotated stimulus (90° for each eye). We examined the difference in decoding accuracy between the original and rotated stimuli for MT and FST in the perspective- and stereoscopic-cue conditions. This manipulation was done to determine if regions encode the retinal motion of the stimulus or the perceptual interpretation. We expected no significant differences in decoding for the stereoscopic stimuli if the regions encode 2D retinal motion but expected differences if they were encoding perceptual information. In contrast, the perspective stimuli preserved 3D motion cues, and thus no change in decoding accuracy was expected. A linear mixed-effect model revealed a significant interaction between the cue type and ROI (*t*_(24)_ = 3.13; *p* = 0.0045), indicating that the effect of the cue type on decoding accuracy depended on the ROI (Fig. 4*C*). Due to the small sample size, rank-sum tests were used to examine if the differences in decoding accuracy between the original and rotated control stimuli were significant. In the perspective-cue condition, there was no significant difference in decoding accuracy for both MT (*p* = 0.16) and FST (*p* = 0.69), suggesting that the rotated stimulus provided sufficient information for discrimination. In the stereoscopic-cue condition, MT also showed no significant difference (*p* = 1), indicating a reliance on the 2D retinal motion of the stimulus. However, FST showed a significant difference (*p* = .031). This suggests that, while preserving equal retinal motion energy, removing the perceptual component of 3D motion reduced decoding performance, indicating that the neural response in FST might reflect the observer's perceptual interpretation of the 3D motion signals.

### Behavioral results, participant classification, and decoding performance between groups

**Figure 5.**
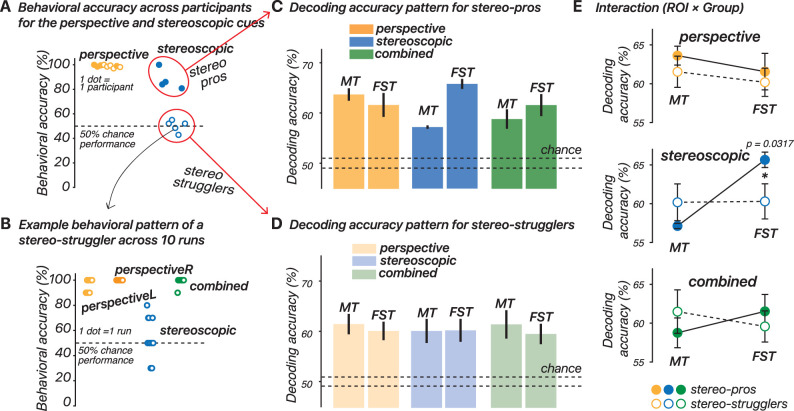
Behavioral results and their effect on decoding performance. ***A***, Behavioral accuracy across participants for the perspective- and stereoscopic-cue conditions. Each dot represents the average behavioral accuracy of an individual participant across 10 runs. Participants were classified into stereo-pros (filled dots) and stereo-strugglers (open dots) based on their performance in the stereoscopic-cue condition. ***B***, Example behavioral pattern of a stereo-struggler across 10 runs. Each run's accuracy is displayed separately for each of the four cue conditions (perspectiveL, perspectiveR, stereoscopic, and combined). ***C***, Decoding accuracy pattern for stereo-pros across three cue conditions (perspective, stereoscopic, combined) and two ROIs (MT and FST). ***D***, Decoding accuracy pattern for stereo-strugglers across the same conditions and ROIs as in ***C***. ***E***, Interaction between ROI (MT vs FST) and group (stereo-pros vs stereo-strugglers) in decoding performance for each cue condition (perspective, stereoscopic, combined). Error bars indicate SEM.

*Behavioral results and participant classification*. We evaluated participants' ability to discriminate motion direction using perspective and stereoscopic cues. Nine instead of ten participants were included in the behavior-related analyses because one participant did not respond in any of the stereoscopic-cue trials. All participants performed well in the perspective-cue condition (98.90% ± 0.32 SE). Interestingly, there was significant variability across participants in the performance in the stereoscopic-cue condition (66.80% ± 6.92 SE). The variability in stereoscopic-cue performance was consistent with prior work (Barendregt et al., 2014, 2016; Thompson et al., 2019; Fulvio et al., 2020). This result prompted the classification of participants into two groups: stereo-pros and stereo-strugglers, based on their stereoscopic-cue performance. Figure 5*A* shows the averaged behavioral accuracy of each participant across 10 runs in the perspective- and stereoscopic-cue conditions. An example of stereo-struggler's performance is shown in Figure 5*B*, with each run's accuracy displayed separately, showing good behavioral performance for conditions with perspective cues but around chance-level (50%) performance in the stereoscopic-cue condition. The stereo-pros, who performed well with stereoscopic cues (87.70% ± 4.24 SE), showed no difference between their performance in the perspective- and stereoscopic-cue conditions. In contrast, stereo-strugglers performed near chance in the stereoscopic-cue condition (50.08% ± 2.08 SE), but their performance in the perspective-cue condition was high (98.62% ± 0.51 *SE*). Mann–Whitney *U* tests confirmed that there was no significant difference between the groups in the perspective-cue condition (*U* = 14.5; *p* = 0.32). Additionally, there was no significant difference in performance between the perspective- and stereoscopic-cue conditions for the stereo-pros (*U* = 12.5; *p* = 0.23). We subsequently performed the analysis separately for stereo-pros versus stereo-strugglers to determine whether the initial pattern of decoding results was masked by averaging over these distinct groups of participants.

*Decoding performance in stereo-pros versus stereo-strugglers*. We analyzed the decoding performance across different cues and ROIs separately for stereo-pros and stereo-strugglers. Figure 5*C* shows the decoding accuracy pattern for stereo-pros, while Figure 5*D* illustrates the pattern for stereo-strugglers. For each group, we examined the decoding accuracy across cue conditions (perspective, stereoscopic, and combined) and ROIs (MT and FST). To test for interactions between ROI and group (stereo-pros vs stereo-strugglers) within each cue condition (Fig. 5*E*), we ran a linear mixed-effect model. In the perspective-cue condition, there was no significant interaction between ROI and group (*F*_(2,14)_ = 0.263; *p* = 0.80). The main effects also showed no significant difference in decoding accuracy between MT and FST (*t*_(14)_ = −1.03; *p* = 0.32) and no significant difference between the groups (*t*_(14)_ = −0.979; *p* = 0.34). For the stereoscopic-cue condition, the model indicated a significant interaction between ROI and group (*F*_(2,14)_ = −3.45; *p* = 0.0039), suggesting that decoding accuracy for FST is particularly influenced by whether the participant was a stereo-pro or a stereo-struggler. A Wilcoxon rank-sum test confirmed that there was a significant difference between stereo-pros and stereo-strugglers for FST (*U* = 29; *p* = 0.032) but not for MT (*U* = 17; *p* = 0.56). For the combined-cue condition, there was no significant interaction between ROI and group (*F*_(2,14)_ = −1.64; *p* = 0.12). There were also no main effects showing significant differences between either ROIs (*t*_(14)_ = 1.30; *p* = 0.22) or groups (*t*_(14)_ = 1.06; *p* = 0.31).

### Effects of coarse-scale bias

*Effects of coarse-scale biases on the interpretation of fMRI decoding results*. Coarse-scale biases, such as radial biases (variations in neural responses that depend on the radial position or direction of stimuli relative to the center of the visual field), origin-of-motion effects (influences on neural activity based on the starting point of motion in a visual stimulus), and edge effects (changes in neural responses caused by the boundaries or edges of visual stimuli), systematically influence neural responses based on retinotopic organization, potentially confounding fMRI measurements (Ritchie et al., 2019). These biases can lead to successful decoding of visual motion and orientation, but significant decoding accuracy does not necessarily indicate genuine neural representation of the target features, as it might instead reflect these systematic biases. For example, motion-decoding accuracies are often greater in V1 than in MT+, contrary to physiological expectations, presumably due to such biases (Kamitani and Tong, 2006; Serences and Boynton, 2007; Beckett et al., 2012; Wang et al., 2014). Initially, our primary analyses focused on MT and FST to streamline the analysis and address the main hypotheses. To align our results with existing literature that extensively examines biases in V1 and MT+, we subsequently included V1 and MST in our analyses. This inclusion allowed us to investigate the relationship between retinotopic properties, BOLD responses, and decoding performance in key ROIs.

*Context for interpreting the results for FST*. While we report retinotopic biases for FST alongside V1, MT, and MST, the results for FST should be interpreted with caution. Monkey FST has very coarse retinotopic organization (Desimone and Ungerleider, 1986; Felleman and Van Essen, 1991; Thompson et al., 2023). Consequently, in humans, the pRF estimates for FST are likely inaccurate due to the region’s large receptive fields, especially relative to the size of our display, as shown by a simulation analysis (Wen et al., 2025). The large receptive fields of FST suggest that retinotopic biases, which are significant in more topographically organized areas like V1, might be less relevant for FST. We have nevertheless included FST in our analysis of retinotopic biases. However, it is crucial to acknowledge that the biases observed in FST may not accurately reflect its true bias due to the limitations of pRF mapping in this region with visual displays of typical size for fMRI experiments.

**Figure 6.**
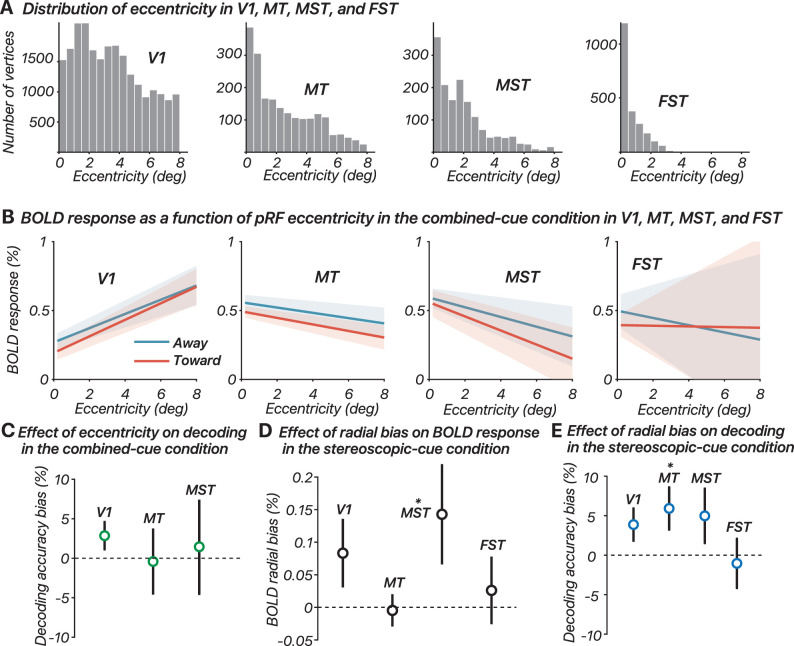
Effects of coarse-scale bias. ***A***, Distribution of eccentricity in V1, MT, MST, and FST. The histograms show the distribution of pRF eccentricity values across different ROIs. ***B***, BOLD response as a function of pRF eccentricity in the combined-cue condition in V1, MT, MST, and FST. Each line represents the best linear fit to the BOLD response as a function of eccentricity for the away (blue) and toward (red) conditions. ***C***, Effect of eccentricity on decoding in the combined-cue condition. The plot shows decoding accuracy bias (%), defined as the percentage difference in decoding accuracy between central (0.2–4°) and edge (4–8°) eccentricities, for different ROIs. ***D***, Effect of radial bias on BOLD response in the stereoscopic-cue condition. The plot illustrates the radial bias in BOLD response for different ROIs, defined as the difference in BOLD response between vertices near the horizontal meridian (HM) and those further away from it (non-HM, NHM). ***E***, Effect of radial bias on decoding in the stereoscopic-cue condition. The plot shows decoding accuracy bias (%), defined as the percentage difference in decoding accuracy between HM and NHM vertices, for different ROIs. Shaded regions and error bars indicate standard errors.

*Effects of eccentricity on BOLD response*. We used the combined-cue stimulus to investigate the origin-of-motion effect. Previous studies suggested that the interaction between motion direction and origin-of-motion can influence BOLD response amplitude, leading to a reversal in preferred motion directions at smaller versus greater eccentricities (Wang et al., 2014). Another potential bias is the edge effect, where the BOLD response increases as a function of eccentricity approaching the stimulus edge. We analyzed data from six participants, who also participated in a population receptive field scan as part of a separate study, examining the relationship between pRF eccentricity and BOLD response in four ROIs: V1, MT, MST, and FST. The pRF eccentricity values of each of the four ROIs are shown in Figure 6*A*. The data were processed to calculate the average BOLD response for both toward and away motion directions. We limited the selection of relevant vertices to those with estimated eccentricities within 0.2–8° and variance explained by the pRF model >5%. We found no obvious differences between toward and away trials in any of the ROIs (Fig. 6*B*). We then used the average response between the two types of motion trials in each ROI for subsequent analyses. We fit a linear mixed-effect model to the data, including random slopes for each participant to account for individual variability in the BOLD response as a function of eccentricity. The analysis was performed both for the overall dataset and separately for each ROI. As expected, the overall results showed a significant effect of eccentricity on BOLD response (*F*_(1,29955)_ = 23.3; *p* < 0.0001; Cohen's *f*² = 0.197), indicating that eccentricity is a significant predictor of BOLD response across all vertices. However, when analyzing each ROI separately, the results varied. In V1, the effect of eccentricity on BOLD response was highly significant (*F*_(1,23898)_ = 37.8; *p* < 0.0001; Cohen's *f*² = 0.319), indicating a strong relationship. In MT, the effect was also significant but much weaker (*F*_(1,2117)_ = 20.5; *p* < 0.0001; Cohen's *f*² = 0.134). In MST, the effect was also significant (*F*_(1,1614)_ = 7.10; *p* = 0.0078; Cohen's *f*² = 0.218), indicating a moderate relationship. In FST, however, the effect of eccentricity on BOLD response was not significant (*F*_(1,2320)_ = 0.0627; *p* = 0.80; Cohen's *f*² = 0.153).

**Movie 1.** Illustration of stimuli used in the experiment. The video shows screen recordings of motion in the toward and away directions for each cue condition: stereoscopic, perspective (left and right), and combined. The left-eye and right-eye views are displayed for crossed fusion. The aperture background was gray, and the area outside was filled with 1/f noise at the plane of fixation, identical for both eyes. [View online]

*Effects of eccentricity on decoding accuracy*. To investigate whether the eccentricity bias observed in the BOLD response translates into a bias in decoding accuracy, we divided the vertices within each ROI into two sub-ROIs based on their eccentricity values. One group focused on the central part of the stimulus with eccentricities between 0.2 and 4°, while the other group focused on the edge part of the stimulus with eccentricities between 4 and 8°. We then performed decoding analysis on these sub-ROIs and calculated the difference in decoding accuracy between the central and edge parts (Fig. 6*C*). We used the Wilcoxon signed-rank test to compare the decoding accuracy between the central and edge parts for each ROI. In V1, the Wilcoxon signed-rank test showed a weak, though not significant, effect indicating that vertices with pRF centers near the center of the stimulus produced greater decoding accuracy than vertices with pRF centers near the edge (*W* = 18; *p* = 0.078), despite the higher BOLD amplitude observed for the edge vertices. In MT and MST, the test indicated no significant difference in decoding accuracy between the two sub-ROIs (MT, *W* = 8; *p* = 0.50; MST, *W* = 11; *p* = 0.50). The FST results are not reported here because, for four out of six participants, we did not have more than two vertices with eccentricity estimates >5°. Reiterating our earlier comment regarding the context for interpreting the results for FST, this is most likely not due to physiological factors but may result from limitations in pRF fitting caused by large receptive fields, receptive field scatter, and the relatively small size of our visual display.

*Effects of radial bias on BOLD response*. The goal of this analysis was to use the BOLD response to the stereoscopic-cue stimuli to investigate the presence of a radial direction bias. Specifically, we aimed to determine whether the BOLD response differed between vertices near the horizontal meridian (HM) and those further away from it (non-HM, NHM). This distinction is crucial because, in the stereoscopic-cue condition, dots move horizontally in each eye, and only at the HM do the dots move radially from the aperture center. If a radial direction bias existed in our design, it should be reflected by differential BOLD responses between vertices with different polar angles. We categorized vertices with polar angles within ±30° of the HM as HM vertices, while those outside this range were classified as NHM vertices. For each ROI—V1, MT, MST, and FST—we calculated the BOLD response separately for HM and NHM vertices. We then computed the difference in BOLD response between HM and NHM vertices, averaged across participants, and plotted these differences for each ROI (Fig. 6*D*). In V1, the mean BOLD response for HM vertices was 0.359% (±0.0964 SE), while for NHM vertices, it was 0.276% (±0.0822 SE). The Wilcoxon signed-rank test indicated that this difference was not statistically significant (*W* = 17.00; *p* = 0.109). In MT, the mean BOLD response for HM vertices was 0.432% (±0.0599 SE), compared with 0.436% (±0.0432 SE) for NHM vertices, and the test also showed no significant difference (*W* = 9.00; *p* = 0.66). For MST, the mean BOLD response for HM vertices was 0.526% (±0.134 SE), while for NHM vertices, it was 0.383% (±0.0893 SE). The Wilcoxon signed-rank test revealed a significant difference in MST (*W* = 19.00; *p* = 0.047), with the *p* value being just below the conventional threshold for significance, indicating the presence of radial bias. Finally, in FST, the mean BOLD response for HM vertices was 0.312% (±0.0707 SE), and for NHM vertices, it was 0.286% (±0.0512 SE), with no significant difference found (*W* = 11; *p* = 0.50). These results indicate that the only significant difference in BOLD response between HM and NHM vertices was found in MST, where a radial direction bias appears to be present. In MT and FST, there were no significant differences, suggesting that the BOLD response in these regions is not significantly influenced by radial direction bias. The weak trend observed in V1 (*p* = 0.11) suggests that there is a potential influence of radial direction bias in this region. To assess whether cortical overrepresentation of the HM (Himmelberg et al., 2022), in combination with the radial bias, could have influenced the decoding results and led to increased BOLD responses for horizontal motion compared with vertical motion, we compared the BOLD response amplitude in V1 between the main and control stereoscopic conditions and found no significant difference (main, 0.317 ± 0.115; control, 0.350 ± 0.109; *p* = 0.609).

*Effects of radial bias on decoding accuracy*. Lastly, we examined whether radial bias had an impact on decoding accuracy by decoding stimulus direction (toward vs away) separately for HM versus NHM vertices and then computed the difference between these two accuracies (Fig. 6*E*). To ensure comparability, we equated the number of vertices in each group by limiting each group to the number of vertices in the group with the smaller count. This was done 500 times to ensure the results did not depend on which vertices were excluded. In V1, the Wilcoxon signed-rank test showed a weak, though not significant, effect indicating that vertices near the HM produced greater decoding accuracy than those further away (*W* = 3; *p* = 0.078), with a mean difference of 3.92% (±2.06 SE). In MT, the test indicated a significant difference in decoding accuracy between the two sub-ROIs (*W* = 2; *p* = 0.047), with vertices near the HM showing better decoding accuracy than those elsewhere, with a mean difference of 5.98% (±2.69 SE). For MST, the results showed no significant difference in decoding accuracy between the HM and NHM vertices (*W* = 5; *p* = 0.16), although there was a trend toward better performance near the HM, with a mean difference of 5.03% (±3.48 SE). In FST, we found no significant difference in decoding accuracy between the HM and NHM vertices (*W* = 12; *p* = 0.66), with a mean difference of −1.05% (±3.15 SE). These results suggest that the radial direction bias observed in the BOLD response does translate into a bias in decoding accuracy in some ROIs. Specifically, MT showed a significant difference, with better decoding accuracy near the HM. V1 and MST showed trends toward better decoding accuracy near the HM, though the effects were not statistically significant. FST showed no significant difference, suggesting that decoding accuracy in this region is not influenced by the radial direction bias.

## Discussion

This study explored the neural mechanisms underlying human 3D motion perception by examining the roles of two key cortical regions—MT and FST. We utilized single-trial fMRI decoding to analyze responses to stereoscopic and perspective cues, focusing on how MT and FST process these cues differently. Our findings indicate that 3D motion direction can be decoded from both regions. However, decoding accuracy based on stereoscopic cues was significantly greater in FST compared with MT, suggesting that FST plays a more prominent role in integrating stereo information. In contrast, decoding accuracy with perspective cues was similar in both regions. Furthermore, in MT, but not FST, there was no appreciable difference in decoding accuracy for stereoscopic 3D motion stimuli compared with rotated control stimuli. Together, these findings suggested that FST encodes perceptual aspects of 3D motion, whereas MT’s decoding performance is more closely linked to the sensory (retinal motion) inputs.

One potential concern is whether the decoding results in FST were influenced by the way the region was localized. We localized FST by contrasting a 2D and 3D motion localizer (Wen et al., 2025). The 2D motion localizer reliably activates MT and MST, but not FST. The 3D motion localizer is a dynamic random-dot stereogram that broadly activates multiple motion-sensitive areas and evokes 3D motion percepts using changing disparity cues only, without coherent 2D motion signals or interocular velocity differences. The difference in activation patterns between the 2D and 3D motion localizers allows FST to be functionally distinguished as a separate region. In contrast to the 3D motion localizer, the 3D stimuli used in the main experiment contained multiple motion signals. The stereoscopic-cue condition contained both changing disparity and interocular velocity differences, along with coherent 2D retinal motion signals. Additionally, the perspective- and combined-cue conditions included perspective cues, which were absent in the 3D localizer. Another key distinction is that in the localizer task, participants performed an unrelated task at fixation and were not required to discriminate motion directions. In contrast, the main experiment required participants to actively report motion direction (toward/away), introducing different perceptual and cognitive demands. Together, these observations suggest that the decoding results in the main experiment are unlikely to be trivially explained by how FST was identified.

Cues to motion perception are a means to an end. Visual perception is full of uncertainty. Utilizing multiple cues to reduce ambiguity is a common strategy in sensory processing, and motion perception is no exception. Both perspective and stereoscopic cues can signal 3D motion direction, but not all human observers utilize these cues equally. In our study, we observed an interesting behavioral pattern: despite using large, high-contrast stimuli with a full second of duration, about half of the participants performed at a chance level in the stereoscopic-cue condition. These participants, who we referred to as the “stereo-strugglers,” showed a significant difference in performance compared with those who could effectively utilize stereoscopic cues, the “stereo-pros.” The underlying neural or perceptual factors influencing the ability to integrate binocular information are not well understood. Indeed, all participants scored well on the Randot Circles Stereotest, and any difference between the stereo-pros (9 ± 0.82) and stereo-strugglers (8.5 ± 1.64) was not statistically significant (*t*_(7)_ = −0.56; *p* = 0.593). A deficit in binocular disparity sensitivity is therefore unlikely to account for impaired stereomotion perception. Studies such as those of Barendregt et al. (2014, 2016) have documented similar phenomena, where impairments in motion-in-depth perception, referred to as stereomotion scotomas, occur in otherwise healthy individuals. These deficits are thought to originate near the stage of binocular integration, where signals from the two eyes are combined. Additionally, Lema and Blake (1977) reported that stereoblind individuals did not exhibit improved visual performance when using both eyes together, indicating a lack of binocular summation. This suggests potential issues in the integration of visual information from the two eyes in these individuals, which could explain why some participants in our study struggled with the stereoscopic-cue condition while performing well with perspective cues. This aligns with earlier findings that individuals who are poor at stereomotion tasks perform well when utilizing perspective or combined cues (Thompson et al., 2019; Fulvio et al., 2020).

The clear pattern of behavioral differences allowed us to investigate whether these variations extended to neural encoding. We analyzed the decoding results separately for stereo-pros and stereo-strugglers, revealing distinct patterns in brain activity. Stereo-pros demonstrated higher decoding accuracy in the FST region for the stereoscopic-cue condition, while stereo-strugglers showed significantly lower decoding accuracy. This difference was observed only in FST and not in MT, and no differences in decoding accuracy were found between the groups in the perspective-cue condition. These results suggest that FST may play a crucial role in binocular integration for 3D motion perception, potentially encoding the percept rather than just the visual cues. The vertical control experiment further supports this interpretation. Using vertically rotated stimuli to remove the 3D motion percept, we found that decoding accuracy in FST was negatively affected, whereas MT's decoding accuracy remained unchanged. However, while our findings suggest the specific role of FST in 3D motion perception, we cannot interpret these decoding results as definitive evidence of neural representation. Decoding performance can sometimes reflect arbitrary differences to which the classifier is sensitive, rather than genuine neural representations of the feature of interest.

Certain aspects of the data restricted our ability to link single-trial decoding to behavior. We observed near-perfect performance in conditions with perspective cues, while half of the participants performed at a chance level in the binocular-cue condition, limiting behavioral variability. While these factors limit the ability to relate single-trial decoding accuracy to behavior, adjusting the task difficulty to facilitate these comparisons is not straightforward, as it must be balanced with maintaining sufficient BOLD SNR for decoding. Indeed, our pilot testing explored alternative stimulus parameters, including peripheral presentation, shorter durations, reduced dot density, and shorter interstimulus intervals, but under these conditions, single-trial decoding did not exceed chance levels. Given these limitations, future studies may benefit from using continuous 360° motion directions in the transverse plane, rather than a binary toward/away task, allowing behavioral variability to be captured as deviations in perceived motion direction rather than a strict correct/incorrect classification.

It is also important to mention that under real-world viewing conditions, the pattern of motion projected onto the retina can have several causes, only some of which are investigated here. Broadly speaking, retinal motion signals can be produced by three types of movement: observer movement, eye movement, and object movement. Our stationary participants fixated while a cluster of dots moving coherently toward or away from their cyclopean eye. Thus, we simulated conditions where object motion was manipulated while holding both eye and observer position constant. It therefore remains unclear whether the ambiguity between these different sources of retinal motion is resolved at the stage of MT and FST.

To address concerns of coarse-scale biases affecting our results, we examined the impact of radial biases, origin-of-motion effects, and edge effects on our decoding outcomes. Criticisms of fMRI studies often suggest that retinotopic biases can explain decoding results, especially in early visual areas (Ritchie et al., 2019). Our analyses revealed significant retinotopic biases in V1, moderate biases in MT and MST, and minimal biases in FST, likely due to the increasingly large receptive fields and less stringent retinotopic organization. We did not find a specific origin-of-motion effect in V1; rather, it varied systematically from small to large eccentricity within the aperture, showing little difference between motion directions, potentially due to our stimulus design. Unlike previous studies that used 2D motion stimuli with coherent patterns that create distinct retinotopic signatures, our study employed binocularly presented motion-in-depth stimuli with dots appearing and disappearing within the aperture, avoiding uniform motion from any edge. Together, we reason that areas such as FST with coarse retinotopy are less prone to systematic retinotopic biases and 3D motion stimuli contain fewer low-level confounds compared with 2D frontoparallel-motion stimuli. Future studies should further refine these analyses and explore methods to distinguish genuine neural representations from systematic biases.

3D motion perception is a complex process that relies on a network of cortical areas, and we do not assume that any single region serves as the sole bridge locus. While MT and FST were the focus of this study, other areas—including V1, MST, V3A, V6, and IPS0—are involved in motion processing. As noted in Results, we included V1 and MST to examine the effects of coarse-scale bias. However, direct comparisons of decoding accuracy between V1 and higher-order motion areas are difficult to interpret since retinotopic biases are expected to have a greater impact on V1 decoding. Likewise, our stimuli were designed to target areas MT and FST and may not have optimally engaged areas such as MST, which prefer much larger and faster-moving stimuli. For the current experiments, these factors would make direct comparisons of decoding accuracy in V1 and MST to MT and FST less reliable. Beyond these areas, V6 has been proposed as a motion-processing candidate due to its strong direction selectivity and evidence of depth-sensitive object and self-motion representations (Galletti and Fattori, 2003; Cardin and Smith, 2011; Fischer et al., 2012; Nau et al., 2018; Pitzalis et al., 2020). V3A and IPS0 have also been implicated in 3D motion processing (Wen et al., 2023). While these areas may play critical roles in 3D motion perception, our current goal was to specifically investigate commonly overlooked area FST and its functional distinction from MT. Future studies should examine the broader cortical network that supports 3D motion perception.

As future studies continue to explore the functional and anatomical homologies of the MT complex across humans and nonhuman primates (NHPs), it will be imperative to consider how the ROIs are defined. In macaques, the anatomical locations of the areas differ markedly across animals (Van Essen et al., 1981) and current anatomically defined ROIs for humans show little overlap with their functionally defined counterparts (Wen et al., 2025). Consequently, how ROIs are delineated is expected to substantially impact conclusions, and this highlights the importance of using functional localizers. Such sources of variability may have contributed to earlier findings of species differences in depth cue integration within MT, even with matched MRI stimulus protocols (Armendariz et al., 2019). Likewise, while a recent MRI study reported potential species differences in connectivity within the MT complex (Ruan et al., 2025), it will be important to validate those results using functionally defined atlases at a minimum and ideally direct anatomical comparison.

The present study aimed to explore the interplay between visual cues, perceptual state, and specific ROIs in the brain. Motion processing begins with the dynamic pattern of retinal stimulation, which through the motion-processing hierarchy is transformed into an interpretation of the movement of objects in the world. Our results suggest that crucial components of this process take place within separate areas of hMT+, highlighting a distinct role of FST, an area that has primarily been studied in NHPs. Our results further suggest that perspective and stereoscopic cues to 3D motion are encoded in functionally distinct areas within the motion complex and, moreover, that both MT and FST are crucially involved in the transformation of retinal signals into a perceptual report.
